# Management strategies for post-transplant IgA nephropathy in kidney transplant recipients: a scoping review

**DOI:** 10.1186/s12882-026-05260-x

**Published:** 2026-08-20

**Authors:** Samuel Bell, Michael Toal, Mark McClure, Alexander P. Maxwell, Gareth J. McKay

**Affiliations:** 1 Centre for Public Health, Queen’s University Belfast, Belfast, UK; 2 Regional Nephrology & Transplant Unit, Belfast City Hospital, Belfast, BT9 7AB UK

**Keywords:** Kidney transplantation, Immunoglobulin a nephropathy, Immunosuppression, Scoping review, Graft failure

## Abstract

**Background:**

Post-transplant Immunoglobulin A Nephropathy (IgAN) is an important cause of premature graft loss. Management strategies are often extrapolated from native IgAN, with available evidence limited to heterogeneous, predominantly small retrospective studies.

**Objectives:**

To characterise diagnostic criteria, map management strategies, and summarise associated clinical outcomes in post-transplant IgAN.

**Methods:**

A literature search was performed in MEDLINE, Embase, Web of Science, and Scopus (1st January 2000–11th December 2025) following Joanna Briggs Institute (JBI) and PRISMA-ScR guidelines. English-language studies of adult kidney transplant recipients with biopsy-proven post-transplant IgAN and documented management strategies were included. Data on study design, cohorts, diagnostic criteria, interventions, and outcomes were charted and narratively described.

**Results:**

Twenty-seven studies met the inclusion criteria. Most were single-centre retrospective cohorts. Diagnostic criteria varied, but typically histological evidence of IgA deposition alone was sufficient. IgAN was often clinically relevant, with proteinuria > 1 g/day frequently reported. Reported treatments included renin-angiotensin-aldosterone system (RAAS)-blockade, immunosuppression adjustment, rituximab, tonsillectomy and pulsed corticosteroids. Several small case series explored emerging therapies (iptacopan, budesonide, telitacicept). Study heterogeneity precluded quantitative data synthesis.

**Conclusions:**

RAAS-blockade was the most commonly used intervention and was associated with benefit in several studies. Tonsillectomy was associated with improved outcomes but reported only in Japanese cohorts. Other interventions (rituximab, pulsed steroids, emerging therapies) warrant prospective clinical trials. Amid evolving paradigms in native IgAN management, this review highlights heterogeneity in diagnostic criteria, outcome reporting, and interventions for the management of post-transplant IgAN. Robust prospective, multicentre studies are urgently required to define optimal management in this high-risk population.

**Supplementary Information:**

The online version contains supplementary material available at https://doi.org/10.1186/s12882-026-05260-x.

## Background

Immunoglobulin A Nephropathy (IgAN) is the most common glomerulonephritis worldwide [1]. Affected individuals have a high lifetime risk of progression to end-stage kidney disease (ESKD) [2], often at a young age, making them ideal candidates for kidney transplantation. Disease recurrence after kidney transplantation is common, with rates reported between 9 and 53% [3]. This is associated with reduced graft survival and premature graft loss [4], adversely affecting long-term patient outcomes.

Advances in the understanding of IgAN pathogenesis, particularly the multi-hit hypothesis involving aberrant IgA1 glycosylation, immune complex formation and subsequent mesangial deposition [5], have driven rapid expansion in targeted therapies for native disease. This includes targeting galactose-deficient IgA1 (Gd-IgA1) production and complement-mediated inflammation [5]. The role of these emerging therapies in post-transplant IgAN remains unclear, where background immunosuppression and competing clinical risk fundamentally alter treatment considerations. Prior systematic reviews and meta-analyses have predominantly focussed on epidemiology, pathophysiology, recurrence risk, or strategies to reduce recurrence after kidney transplantation; reviews on management are largely narrative and non-systematic [6–10]. Consequently, there is no consensus management strategy post-transplantation, and treatment decisions are guided by evidence in native disease and small observational studies in transplant recipients.

Other key aspects of post-transplant IgAN remain poorly defined. The aetiology of ESKD may be unknown (due to lack of diagnostic native biopsy), and marked heterogeneity exists in transplant biopsy practice and histological thresholds for diagnosis. This includes whether IgA deposition alone is sufficient for the diagnosis, or whether accompanying clinical or histological features are required. The post-transplant IgAN literature mainly consists of small, heterogeneous, observational studies, limiting quantitative synthesis but lending itself to systematic mapping within a scoping review. As such, the aims of this post-transplant IgAN scoping review are:

1. Characterise how post-transplant IgAN is defined.
2. Identify management strategies reported for post-transplant IgAN.
3. Summarise reported clinical outcomes and adverse events.

## Methods

The study review protocol was developed in accordance with guidelines from the Joanna Briggs Institute (JBI) [11] and adheres to the methodological framework by Arksey and O’Malley [12]. Following initial screening and extraction, the protocol was registered with the Open Science Framework (OSF, https://doi.org/10.17605/OSF.IO/K7RUJ) and completed in accordance with the PRISMA extension for scoping reviews [13]. No deviations from the OSF protocol occurred.

### Study definitions

#### Immunoglobulin A Nephropathy (IgAN)

a primary, immune-mediated proliferative glomerulonephritis characterised by IgA mesangial deposits.

#### Kidney Transplantation

an allogeneic, human kidney transplantation.

#### Post-Transplant Immunoglobulin A Nephropathy (Post-Transplant IgAN)

in this review, all post-transplant IgAN was considered, regardless of the aetiology of the primary kidney disease.

#### Management

any documented intervention following diagnosis of post-transplant IgAN, including modification of immunosuppression, antiproteinuric therapies, supportive measures (for example, blood pressure management) or disease-specific therapies.

#### Outcomes

any reported clinical or graft-specific outcomes following treatment e.g. reduction in proteinuria, trajectory of graft function, graft survival.

### Search strategy

Databases included MEDLINE ALL, Embase, Web of Science and Scopus. A comprehensive search strategy was formulated by the research team, in collaboration with a medical librarian. Search strategies were developed using three core concepts: IgA nephropathy, kidney transplantation, and recurrence. This was expanded using relevant controlled vocabulary (e.g. Medical Subject Headings), associated synonyms, and the Boolean operators AND and OR. The search was performed on December 11th, 2025. A date limit was applied to include studies published from January 1st, 2000, until the search date, and results were restricted to those written in English. Full eligibility criteria are outlined in Table 1.

Citations identified were exported to Covidence systematic review software (Veritas Health Innovation, Melbourne, Australia. Available at http://www.covidence.org) and duplicates were identified and removed.

Exact search strategies for all databases are available in Supplementary Tables 1–4.

### Eligibility criteria for post-transplant IgAN

**Table 1:** Inclusion and exclusion criteria for post-transplant IgAN

Criteria Type	Inclusion	Exclusion
Population	• Kidney transplant recipients • Biopsy-proven post-transplant IgAN • Age > 18 years at diagnosis (mixed cohorts eligible if adult data predominantly represented)	• Mixed native/transplant cohorts without separable data • Predominant paediatric cohorts • Mixed transplant cohorts with other diseases without separable IgAN data
Interventions	Any intervention, including: • Immunosuppression adjustment / addition • Antiproteinuric therapies • Other supportive or disease specific therapies	• No extractable management data • No extractable clinical outcomes • Single patient interventions
Types of Sources	• Case series (*n* ≥ 2 patients) • Cohort studies (retrospective/prospective) • Randomised controlled trials	• Reviews, editorials, commentaries without extractable data • Single case reports • Conference abstracts / posters
Time Period	January 1st, 2000, to December 11th, 2025	
Language	English	

Eligibility criteria were designed to maximise the capture of clinically relevant post-transplant IgAN while excluding major confounders. Recurrent and *de novo* post-transplant IgAN were considered together. This was due to inconsistent classification across the literature and a frequent lack of confirmatory native kidney diagnosis, which limited distinction across multiple cohorts. Studies with sufficient treatment cohorts (*n* ≥ 2 patients) were included. If a study met inclusion criteria but contained single patient subgroups, these subgroups were excluded. Grey literature (e.g. conference abstracts) was excluded given limited peer review and insufficient data for extraction.

### Study screening and selection

Following duplication removal, title and abstract screening was performed independently by two reviewers (S.B and M.T). All titles and abstracts were screened by S.B. M.T independently screened a proportion of studies to ensure reviewer calibration through two sequential 10% calibration exercises. Agreement was high in both rounds (94.4% and 94.9%). Initially, Cohen’s Kappa was low (0.31), reflecting the low prevalence of included studies (and high prevalence of exclusions). Following reviewer calibration, Cohen’s Kappa increased to 0.60, indicating improved agreement. Disagreements were resolved through discussion between S.B and M.T and additional reviewer (A.P.M) before final screening. Full text review was then carried out by S.B.

### Data charting

A data charting tool was developed by the research team. The following data items were extracted from the included articles: title, year, journal, author(s), country of origin, study design, cohort characteristics, diagnostic criteria, management, and outcomes.

### Data summary

Data is presented in narrative form by three key themes: study design and baseline characteristics; diagnostic criteria; and management strategies and observed outcomes. Studies were not formally assessed for risk of bias, in keeping with standard scoping review methodology. Findings are presented to map the current available evidence. Results are presented narratively, and due to significant heterogeneity, were not pooled statistically. Where studies reported concurrent or overlapping interventions, these were extracted as described within the original studies. Interpretation was cautious, recognising the attribution of observed outcomes to individual therapies was often not possible.

## Results

**Fig. 1:**
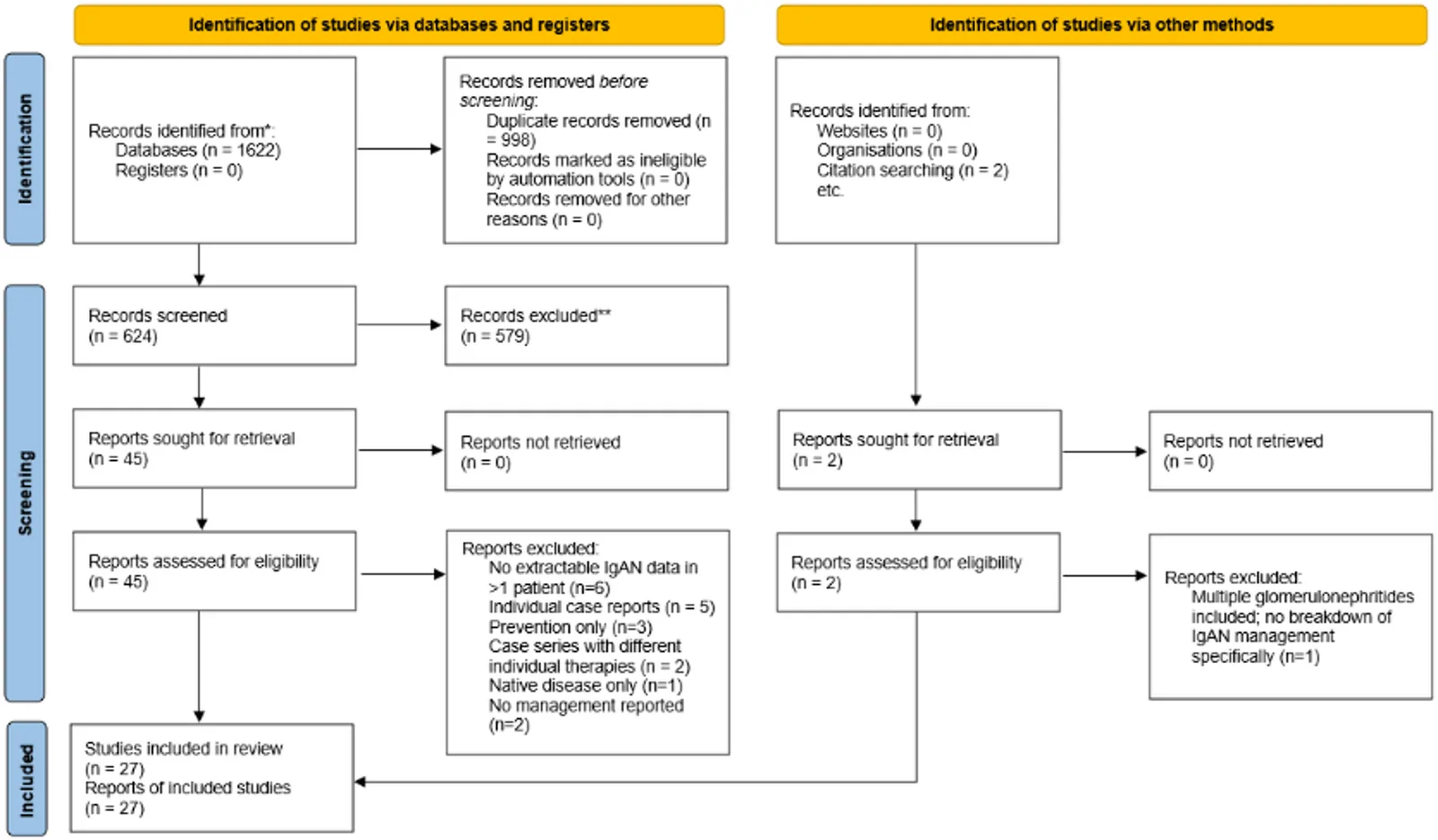
PRISMA flow diagram summarising the study selection process for post-transplant IgAN publications

Database searching identified 1622 articles. After deduplication, 624 potential publications were screened. One additional study was identified via citation searching. A final total of 27 articles met pre-specified inclusion criteria. The reasons for exclusion are outlined in the PRISMA flow chart [14] provided in Fig. 1.

### Study design and baseline characteristics

**Table 2:** Study design and baseline characteristics at diagnosis for entire post-transplant IgAN cohort (unless otherwise stated). Units are reported as stated in original manuscript. Weighted averages were calculated within individual studies, where available, to provide descriptive baseline summaries

Study	Intervention / Intervention Categories	Country of Origin	Study Design	Post-Transplant IgAN Cohort (*n*)	Intervention Subgroups (*n*)	Comparator Group?	Confirmed Native IgAN (%)	Baseline Proteinuria	Baseline Creatinine or eGFR
Oka 2000 [38]	RAAS-Blockade	Japan	Retrospective Single-Centre Cohort (Prospective Intervention Arm)	21	10	No	0%	1.2 g/day	1.5 mg/dL
Jeong 2003 [15]	RAAS-Blockade	South Korea	Retrospective Single-Centre Cohort	54	14	No	16.7%	2.1 g/day **	1.3 mg/dL **
Namba 2004 [16]	RAAS-Blockade	Japan	Retrospective Single-Centre Cohort	24	18	Yes	100%	0.72 g/day † (*n* = 20)	1.69 mg/dL†
Jeong 2004 [17]	Immunosuppression	South Korea	Retrospective Single-Centre Cohort	71	Multiple	Yes	12.7%	Crescentic: 3.4 g/day Non-Crescentic: 1.5 g/day	Crescentic: 2.0 mg/dL Non-Crescentic: 1.6 mg/dL
Chandrakantan 2005 [18]	RAAS-Blockade; Immunosuppression	USA	Retrospective Single-Centre Cohort	20	Multiple	Yes	100%	3.0 g/day †	NR
Courtney 2006 [19]	RAAS-Blockade	UK	Retrospective Single-Centre Cohort	13	9	Yes	100%	NR	NR
Mousson 2007 [20]	Pulsed Steroids	France	Retrospective Single-Centre Cohort	22	2	No	100%	0.8–2.4 g/day	NR
Tang 2008 [21]	Immunosuppression; Corticosteroid-based strategies	China	Retrospective Single-Centre Case Series	10	Multiple	No	50%	2.06 g/day	3.37 mg/dL
Kiattisunthorn 2008 [22]	RAAS-Blockade; Corticosteroid-based strategies; Immunosuppression	Thailand	Retrospective Single-Centre Cohort	30	Multiple	Yes	3.3%	3.1 g/day	2.86 mg/dL
Ushigome 2009 [23]	Tonsillectomy	Japan	Retrospective Single-Centre Case Series	4	4	No	100%	Dipstick 0–3+	1.85 mg/dL†
Kennoki 2009 [24]	Tonsillectomy	Japan	Retrospective Single-Centre Cohort	63	16	Yes	100%	0.79 g/day †	1.24 mg/dL †
Koshino 2013 [25]	Tonsillectomy	Japan	Retrospective Single-Centre Case Series	7	7	No	100%	Dipstick 0–3+	1.65 mg/dL
Hotta 2013 [26]	Tonsillectomy; Corticosteroid-based strategies	Japan	Retrospective Single-Centre Cohort	15	Multiple	No	60%	0.53 g/g	1.34 mg/dL †
Wang 2014 [27]	RAAS-Blockade; Tonsillectomy; Corticosteroid-based strategies	Japan	Retrospective Single-Centre Case Series	5	Multiple	No	NR*	0.78 g/day	1.22 mg/dL
Messina 2016 [28]	Corticosteroid-based strategies	Italy	Retrospective Single-Centre Cohort	29	16	Yes	37.9%	1.67 g/day †	1.9 mg/dL †
Chancharoenthana 2017 [37]	Rituximab	Thailand	Retrospective Single-Centre Case Series	3	3	No	100%	4.8 g/day†	1.25 mg/dL
Nihei 2017 [29]	Tonsillectomy	Japan	Retrospective Single-Centre Cohort	12	5	Yes	100%	0.18 g/g †	1.23 mg/dL †
Cordeiro Cabral 2018 [30]	RAAS-Blockade; Statin; Immunosuppression; Corticosteroid-based strategies; Cyclophosphamide	Brazil	Retrospective Single-Centre Cohort	47	Multiple	Yes	6.4%	3.4 g/g	*43.6 ml/min/1.73 m* ^*2*^
Matsukuma 2018 [31]	Corticosteroid-based strategies	Japan	Retrospective Single-Centre Cohort	7	7	No	16.7%	0.82 g/g	*48.7 ml/min/1.73 m* ^*2*^
Park 2019 [40]	RAAS-Blockade; Anti-hypertensives	South Korea	Retrospective Bi-Centre Cohort	464	Multiple	Yes	21.6%	Dipstick 1–3+	1.70 mg/dL†
Chancharoenthana 2021 [32]	Rituximab	Thailand	Retrospective Single-Centre Cohort	64	21	Yes	NR	4.48 g/day	2.1 mg/dL
Uffing 2021 [41]	RAAS-Blockade	Multiple	Retrospective Multicentre Cohort	108	78	Yes	75.9%	NR	NR
Lopez-Martinez 2022 [33]	Budesonide	Spain	Retrospective Single-Centre Case Series	5	5	No	NR	PCR 1.5, units not reported (assumption is g/g)	NR
Gandolfini 2023 [34]	Budesonide	Italy	Retrospective Single-Centre Case Series	10	10	No	NR	NR	NR
Ganesh 2024 [39]	RAAS-Blockade; Rituximab; Corticosteroid-based strategies; Apheresis	India	Prospective Single-Centre Cohort	64	Multiple	No	20.3%	Provided for “pathogenic” IgA (*n* = 46) 1.2 g/day	Provided for “pathogenic” IgA (*n* = 46) 1.84 mg/dL
Sanna 2025 [35]	Iptacopan	Italy	Retrospective Single-Centre Case Series	5	5	No	NR*	2.36 g/day†	1.88 mg/dL†
Xu 2025 [36]	Telitacicept	China	Retrospective Single-Centre Case Series	10	10	No	NR*	2.92 g/day	141.9 µmol/L

* Not explicitly reported, but paper references “recurrent IgAN”

** Only interventional cohort reported

† Weighted averages from provided data

Abbreviations: Aza, azathioprine; eGFR, estimated glomerular filtration rate; MMF, mycophenolate mofetil; NR, not recorded; PCR, protein: creatinine ratio; RAAS, renin angiotensin aldosterone system

Study design and baseline characteristics are outlined in Table 2. Most studies were retrospective and single-centre [15–37], with two studies reporting a prospective component [38, 39]. There was one bi-centre study [40], and one multinational, multicentre study spanning three continents [41] from the Post-Transplant Glomerular Disease (TANGO) study [42]. Asian cohorts were predominantly represented (*n* = 18), with cohorts from Japan (*n* = 9), South Korea (*n* = 3), Thailand (*n* = 3), China (*n* = 2) and India (*n* = 1). Other studies originated from Europe (*n* = 6), North America (*n* = 1, USA) and South America (*n* = 1, Brazil). Cohort sizes ranged from 3 to 464 patients, often with smaller intervention subgroups. Many studies did not contain comparators. Most studies included a predominance of living donor kidney transplants and male recipients – male recipients were the majority sex in 22 studies (Supplementary Table 5).

Disease severity at diagnosis (where reported) was varied but often clinically significant; most cohorts had mean proteinuria exceeding 1 g/day. Baseline serum creatinine was typically < 2 mg/dL, although severe presentations with rapidly progressive glomerulonephritis (RPGN) were reported.

Typically, maintenance immunosuppression was calcineurin-inhibitor based, with or without antiproliferative agents and corticosteroids. Earlier cohorts predominantly utilised ciclosporin and azathioprine, with later cohorts utilising tacrolimus and mycophenolic acid derivatives, reflecting the evolution of modern immunosuppression practices (Supplementary Table 5).

Where reported, the proportion of confirmed native IgAN was extracted from each study. This confirms recurrent IgAN post-transplantation, rather than *de novo* IgAN. However, we did not consider the absence of confirmed native IgAN diagnosis (due to a lack of native kidney biopsy, or lack of reporting) as sufficient to classify post-transplant IgAN as *de novo*. This lack of distinction across heterogeneous sample sizes, overlapping therapies, and other confounders, prevented meaningful distinction and quantitative assessment between recurrent and *de novo* IgAN.

In nine studies [16, 18–20, 23–25, 29, 37], all transplant recipients had confirmed native IgAN. Three studies [27, 35, 36] described post-transplant IgAN as recurrent but did not explicitly confirm the native kidney diagnosis. Across remaining studies, rates of confirmed native IgAN varied or was not reported.

### Diagnostic criteria

**Table 3:** Diagnostic criteria for post-transplant IgAN

Study	Biopsy Indication (*n* – where reported)	Time from Transplant to Diagnosis (months)	Histological Diagnostic Criteria	Histological Grading	Additional Diagnostic Criteria
Oka 2000	Proteinuria (17) Non-episode (4)	76.8 ± 44.3*	Dominant mesangial IgA deposition	Glomerular changes (0–3), Interstitial Fibrosis (0–3), Proportion of glomeruli with: obsolescence, crescents, segmental sclerosis, adhesions (%)	NR
Jeong 2003	Graft dysfunction (14) Proteinuria (22) Microscopic Haematuria (18)	49.6 ± 23.2	Dominant mesangial IgA deposition *≥* 6 glomeruli in specimen	Proportion of glomeruli with global and segmental sclerosis (%), IFTA (0–3) Vascular Intimal Thickening (0–3)	NR
Namba 2004	Proteinuria (16) Non-episode (5) Protocol (3)	7.2 ± 47.9*	Dominant mesangial IgA deposition	NR	NR
Jeong 2004	Graft dysfunction (19) Proteinuria (30) Isolated Haematuria (22)	59.5 ± 41.7 in Crescentic Group 59.4 ± 27.4 in non-Crescentic Group	Dominant mesangial IgA deposition *≥* 6 glomeruli and *≥* 1 intralobular artery in specimen	Degrees of interstitial inflammation, tubulitis, IFTA, arteriolar hyalinosis and vascular intimal thickening were scored from 0–3 (in accordance with Banff) Intensity and extent of mesangial immunoglobulin / complement staining scored (0–3)	NR
Chandrakantan 2005	Graft dysfunction and/or Proteinuria	97.2 ± 39.6* (Aza) 42.0 ± 32.4* (MMF)	Dominant mesangial IgA deposition	NR	NR
Courtney 2006	Graft dysfunction and/or Proteinuria	NR	Dominant mesangial IgA deposition with “glomerular changes”	NR	NR
Mousson 2007	Rapidly Progressive Glomerulonephritis	NR	NR	NR	Presence of crescents
Tang 2008	Rapidly Progressive Glomerulonephritis	Years provided in 0.5 increments. Average is 5 years post-transplant (3–7).	Dominant mesangial IgA deposition	Degree of crescents and sclerosis quantified (%) IgA deposition, proliferation and IFTA (1–3+)	Presence of crescents
Kiattisunthorn 2008	Graft dysfunction, Proteinuria, Haematuria (or a combination)	33.6 (median)	Dominant mesangial IgA deposition with mesangial proliferation	IFTA (mild < 25%, moderate 25–50%, severe > 50%) Presence of extra-capillary proliferation, fibrinoid necrosis, or glomerulosclerosis	NR
Ushigome 2009	Proteinuria and/or Haematuria	48.6*	Dominant mesangial IgA deposition	Katafuchi et al. glomerular score	NR
Kennoki 2009	Protocol	NR, however, protocol biopsies done at 1, 12 and 24 months	Dominant mesangial IgA deposition with at least mild mesangial changes	Degree of mesangial proliferation graded (minor, focal proliferative and diffuse proliferative)	“Donor derived” IgA transmission excluded by pre-implant biopsy
Koshino 2013	Proteinuria and/or Haematuria	Time to urinary findings provided rather than biopsy: 40.3 ± 31.2	Dominant mesangial IgA deposition	Katafuchi et al. glomerular score	NR
Hotta 2013	Protocol (14) Graft dysfunction (1)	44.1 ± 27.1	Dominant mesangial IgA deposition	Japanese histological grading system	NR
Wang 2014	Proteinuria	Time to urinary findings: 19.8 ± 18.6 Biopsy performed 3–5 months following this	Dominant mesangial IgA deposition	Banff criteria	NR
Messina 2016	Graft dysfunction and/or Proteinuria	46.6 (4.8-136.8) in Intervention group 99.0 (10.1-172.2) in Control group	Dominant mesangial IgA deposition	MEST where biopsy available and adequate (*n* = 23)	NR
Chancharoenthana 2017	Proteinuria (1) Nephrotic syndrome (1) Protocol (1)	3.1 years, 8.4 years, and 12.5 years (*n* = 3)	NR	NR	NR
Nihei 2017	Protocol	37.4 ± 25.7	Dominant mesangial IgA deposition	Banff and MEST	”Donor derived” IgA transmission excluded by pre-implant biopsy, new haematuria and/or proteinuria
Cordeiro Cabral 2018	Graft dysfunction and/or Proteinuria	49.1 ± 32.7	Dominant mesangial IgA deposition	*if >* 8 glomeruli in specimen then MEST calculated	Clinically relevant IgAN where urinary Protein: Creatinine Ratio > 1.0 g/g or allograft dysfunction present
Matsukuma 2018	Mix of “for cause” or “protocol”	79.2 ± 56.4*	Dominant mesangial IgA deposition	MEST	NR
Park 2019	Mix of “for cause” or “protocol”	53.9†	Dominant mesangial IgA deposition	MEST-C where biopsy available and adequate	NR
Chancharoenthana 2021	Mix of “for cause” or “surveillance”	64.2 (median), 38.1–162.4 (IQR)	NR	MEST-C	NR
Uffing 2021	Protocol (5%) Clinically indicated (95%)	40.8*	Dominant mesangial IgA deposition	NR	NR
Lopez-Martinez 2022	NR	NR	NR	NR	NR
Gandolfini 2023	NR	98.8 ± 94.9	NR	Banff	NR
Ganesh 2024	Graft dysfunction and/or Proteinuria	Given as breakdown: - 56.5% within 1 year with 30.4% total within 1 month of transplant (only 1 with Pathogenic IgA Deposition) - 43.4% after 1 year	Dominant mesangial IgA deposition	MEST-C	Biopsies split into “Pathogenic IgA deposits” (*n* = 46) or “Incidental” (*n* = 18) Pathogenic IgAN defined as: - Active IgA lesions - Proteinuria - IgA with Acute Tubular Injury
Sanna 2025	Graft dysfunction and/or Proteinuria	31.8*	NR	MEST-C reported (*n* = 2) IgA, C3, and C5b-9 graded in all	NR
Xu 2025	NR	60 ± 31.2*	NR	MEST-C reported (*n* = 8)	Secondary IgAN excluded

* converted from years

† Weighted averages from provided data

Abbreviations: Aza, azathioprine; IFTA, interstitial fibrosis and tubular atrophy; IgA, Immunoglobulin A; MEST(-C), Oxford classification system for assessing IgAN histologically. M: Mesangial hypercellularity, E: Endocapillary proliferation, S: Segmental sclerosis/adhesion, T: Tubular atrophy/ interstitial fibrosis, C: Crescents; MMF, mycophenolate mofetil; NR, not reported

Kidney transplant biopsy indications were reported in 24 studies (Table 3). The majority were for clinical indications such as proteinuria and graft dysfunction, while 10 studies included protocol biopsies.

All studies required histological confirmation of post-transplant IgAN, although diagnostic criteria were inconsistently reported. Many studies defined post-transplant IgAN solely by the presence of IgA deposition, whereas only three studies additionally mandated glomerular abnormalities alongside IgA deposition [19, 22, 24]. In several reports, additional histological or clinical criteria were required to proceed to an intervention.

The average time to diagnosis of post-transplant IgAN was highly variable and likely related to local biopsy practices. A delay between the onset of clinical features and biopsy timing was frequently implied but rarely quantified. A range of histological grading tools were employed, with recent studies typically incorporating the Oxford Classification of IgA Nephropathy (MEST-C) scoring system.

### Management strategies and observed outcomes

**Table 4:** Management strategies and observed outcomes for post-transplant IgAN

Study	Management	Sample (*n*)	Additional Eligibility (for Intervention)	Specific Therapy	Comparator Intervention	Outcome
Oka 2000	RAAS-blockade	10	Stable graft function Proteinuria 0.3–3 g/day Moderate to severe hypertension Renal artery stenosis excluded	Trandolapril 1 mg OD	Nil	67% proteinuria reduction, stable allograft function
Jeong 2003	RAAS-blockade	14	Proteinuria > 0.5 g Treatment duration > 12 months Superimposed Glomerulonephritis / Transplant glomerulopathy not present	Enalapril (dose not provided)	Nil	9/14 patients with > 25% proteinuria reduction Presence of segmental sclerosis predicted poorer response
Namba 2004	RAAS-blockade	18	NR	Any RAAS-blockade	No RAAS-blockade (*n* = 6)	Use of RAAS-blockade at time of biopsy associated with lower risk of graft loss (2/18 in treatment group, 2/6 in control)
Chandrakantan 2005	RAAS-blockade	13	NR	Any RAAS-blockade		Ineffective (only 1/13 patients had > 25% proteinuria reduction)
Courtney 2006	RAAS-blockade	9	Treatment duration > 12 months	Any RAAS-blockade	No RAAS-blockade (*n* = 4)	At latest follow-up: 3/9 patients on RAAS-blockade had returned to dialysis vs. all (4/4) patients not on RAAS-blockade, but follow-up times and graft survival not provided
Kiattisunthorn 2008	RAAS-Blockade	13	NR	Any RAAS-blockade	Other subgroups	No difference observed in graft loss between patients on RAAS-blockade or not
Wang 2014	RAAS-Blockade	5	NR	Any RAAS-blockade	Nil	Pre-tonsillectomy, RAAS-blockade alone reduced proteinuria by 49% (0.78 g/day to 0.4 g/day)
Cordeiro Cabral 2018	RAAS-Blockade	42	NR	Any RAAS-blockade	Other subgroups	No effect in univariate analysis
Park 2019	RAAS-Blockade	38	NR	Any RAAS-blockade	Other subgroups (including no therapy)	Single agent RAAS-blockade demonstrated better death-censored graft survival compared to combination therapy or alternative antihypertensive therapy alone. No therapy had the best prognosis (*n* = 272).
	Calcium Channel Blocker and/or Beta Blocker	33	NR	Any Calcium Channel Blocker / Beta-blocker		
	Combination	121	NR	Any RAAS-blockade with combination Calcium Channel Blocker and/or Beta-blocker		
Uffing 2021	RAAS-Blockade	78	NR	Any RAAS-blockade	No RAAS-blockade (*n* = 30)	No difference in death censored graft survival
Cordeiro Cabral 2018	RAAS-Blockade (Dual)	21	NR	Any combination	Other subgroups	Trend towards improved graft survival in univariate analysis (*p* = 0.07) but no difference in a multivariate analysis (*p* = 0.4)
Ganesh 2024	RAAS-Blockade and Corticosteroid-based strategies	38	IgA deposits with or without acute tubular injury, none of outlined “active” lesions	RAAS-blockade: Any Increase to Oral Prednisolone with a “prolonged tapering regimen over 3 months”	Nil	60% and 65% reduction in proteinuria in patients with or without acute tubular injury (respectively)
Jeong 2004	Baseline Immunosuppression	2	Crescentic post-transplant IgAN	Addition of Mycophenolate	Other subgroups	Of the Crescentic patients (*n* = 10), 90% had failed by follow-up. “Increased immunosuppression was ineffective”
		2		Addition of Azathioprine		
Chandrakantan 2005	Baseline Immunosuppression	9	NR	Switching Azathioprine to Mycophenolate	Remained on Azathioprine (*n* = 4) or Mycophenolate (*n* = 7)	Survival analysis showed no difference between the 3 groups
Tang 2008	Baseline Immunosuppression	5	NR	Switching Azathioprine to Mycophenolate	Nil	No between group analysis done, but graft failure occurred in 90% of patients (6/10 by Year 1) despite any therapy.
		5		Switching Ciclosporin to Tacrolimus		
Kiattisunthorn 2008	Baseline Immunosuppression	4	NR	Addition of Mycophenolate	Other subgroups	Immunosuppressive regimen change did not influence graft survival
		2		Switching Azathioprine to Mycophenolate		
Cordeiro Cabral 2018	Baseline Immunosuppression	9	NR	Switching Azathioprine to Mycophenolate	Other subgroups	No effect in univariate analysis
		40	NR	Not Otherwise Specified		No effect in univariate analysis
Mousson 2007	Corticosteroid-based strategies	2	Crescentic IgAN	Methylprednisolone	Nil	Despite pulsed steroids, graft failure occurred at 2 and 27 months respectively
Tang 2008	Corticosteroid-based strategies	10	Crescentic IgAN	Methylprednisolone 500 mg OD for 3 days	Nil	No between group analysis done, but graft failure occurred in 90% of patients (6/10 by Year 1) despite any therapy.
Kiattisunthorn 2008	Corticosteroid-based strategies	10	NR	Methylprednisolone	Other subgroups	Pulsed steroid did not influence graft survival
Messina 2016	Corticosteroid-based strategies	16	Glomerulosclerosis < 50% and 1 or more of the following: - Proteinuria >1 g / day (at biopsy) - Crescents - Rapidly progressive glomerulonephritis	Methylprednisolone 500 mg OD for 3 days, start of months 1,3,5 Oral Prednisolone 0.5 mg/kg alternative days, for 6 months	No pulsed steroids (*n* = 13) but a variety of other treatments trialled	Lower proteinuria and better allograft function observed in steroid treated group. Less patients in intervention group reached a composite outcome of graft loss or doubling of serum creatinine (2/16 vs. 5/13) but this was not statistically significant
Cordeiro Cabral 2018	Corticosteroid-based strategies	30	NR	Methylprednisolone 500–1000 mg OD, for > 3 days (cumulative average 4.1 g)	Other subgroups	No effect in univariate analysis
		39	NR	Increasing Oral Prednisolone: High dose > 0.4 mg/kg for 5.5 mo +/- 5.0 Low dose < 0.4 mg/kg for 18.8 mo +/- 25.8		Significantly higher risk of graft loss in univariate analysis, however association disappeared in multivariate analysis
Matsukuma 2018	Corticosteroid-based strategies	7	Proteinuria > 0.5 g/g creatinine, with any haematuria Cellular or fibrocellular crescents No contraindications to steroid therapy	Methylprednisolone 500 mg OD for 3 days, start of weeks 1 and 2 Oral Prednisolone 20 mg OD, for 6 months then taper to 5 mg OD	Nil	68% reduction proteinuria with stable allograft function
Chancharoenthana 2017	Rituximab	3	NR	Rituximab 375 mg/1.73m^2^ monthly, for 4 months	Nil	By 6 months, all patients had a reported > 40% reduction proteinuria
Chancharoenthana 2021	Rituximab	21	Age 18-70 Proteinuria > 1 g/day (despite 2 months of stable RAAS-blockade) Excluded if no endocapillary hypercellularity, contra-indications for Rituximab, or previous Rituximab treatment	Rituximab 375 mg/1.73m^2^ monthly, for 4 months	Yes, *n* = 43, standard of care (RAAS-blockade and target BP < 130/80)	Lower proteinuria in Rituximab group by 12 months (with 8 cases of complete remission) Improved 24-hour Creatinine Clearance in Rituximab group by 18 months Better allograft survival compared to controls
Ganesh 2024	Rituximab and Corticosteroid-based strategies	4	Active “Pathogenic” IgA deposits, i.e. endocapillary proliferation, crescents, vasculitis	Rituximab 375 mg/m^2^, 2 doses 4 weeks apart Methylprednisolone	Nil	At 1 year, a 55% reduction in proteinuria with stable graft function was observed.
	Rituximab, Corticosteroid-based strategies and Apheresis	4	Active “Pathogenic” IgA deposits, i.e. endocapillary proliferation, crescents, vasculitis	Rituximab 375 mg/m^2^, 2 doses 4 weeks apart Methylprednisolone 500 mg OD for 3 days Apheresis (exchange and number of sessions not provided)		Unclear if apheresis added benefit in addition to Pulsed steroids and Rituximab
Cordeiro Cabral 2018	Cyclophosphamide	8	NR	Cyclophosphamide	Other subgroups	No effect in univariate analysis
Ushigome 2009	Tonsillectomy	4	NR	Tonsillectomy	Nil	Stable allograft function and resolution of dipstick abnormalities
Kennoki, 2009	Tonsillectomy	16	Persistent urinary protein excretion > 0.3 g/day despite RAAS-blockade.	Tonsillectomy	Refused tonsillectomy (*n* = 12)	68% proteinuria reduction with stable allograft function in tonsillectomy group. Control group had no change in proteinuria and a slight rise in Creatinine (1.3 mg/dL to 1.5 mg/dL)
Koshino, 2013	Tonsillectomy	7	NR	Tonsillectomy	Nil	3 patients had complete resolution of dipstick findings (however authors felt in 1 case, it was more related to blood pressure control and weight loss). 2 patients progressed rapidly and lost their graft despite tonsillectomy.
Hotta, 2013	Tonsillectomy	7	NR	Tonsillectomy	Other subgroups	Resolution of microscopic haematuria and reduction in proteinuria On repeat biopsy: • 10/15 had improved histology • 3/15 had stable histology • 2/15 had worse histology The addition of steroid therapy to tonsillectomy had no effect on proteinuria or histological findings.
Hotta, 2013	Tonsillectomy and Corticosteroid-based strategies	8	NR	Tonsillectomy Methylprednisolone 500 mg OD for 3 days	Other subgroups	
Wang, 2014	Tonsillectomy	2	Persistent proteinuria despite 6 months treatment with RAAS-blockade	Tonsillectomy	Nil	No breakdown between subgroups, but a further reduction in proteinuria was seen across all patients (0.4 g/day to 0.06 g/day)
	Tonsillectomy and Corticosteroid-based strategies	3	In addition to above: - Proteinuria > 1 g (*n* = 1) - Persistent proteinuria at 1 year (*n* = 1) - Active endothelial proliferation or cellular crescents (*n* = 1)	Tonsillectomy Methylprednisolone 500 mg OD for 3 days		
Nihei, 2017	Tonsillectomy	5	NR	Tonsillectomy	Refused tonsillectomy (*n* = 7)	In tonsillectomy group: stable proteinuria, stable histology (at 5 years) In control group: significantly worse proteinuria (compared to baseline), worse histology
Sanna, 2025	Iptacopan	5	Failed other therapies	Iptacopan	Nil	3/5 patients had an improvement in proteinuria by 6 months, however only 1 was sustained by 12 months. Allograft function improved or stabilised in 3/5 patients. Repeat kidney biopsy demonstrated a reduction in C3, and C5b-9 deposition
Xu, 2025	Telitacicept	10	Aged between 18 and 70 years; Blood pressure maintained below 140/90 mmHg; Exclusion: Liver failure, heart failure, multiple organ transplants, non-adherence, nephrotic syndrome, crescentic nephritis (> 50%), minimal change disease	Telitacicept 160 mg SC weekly initially, increasing to 240 mg SC weekly on a case-by-case basis	Nil	Proteinuria reduction observed at 3, 6 and 9 months (with 1 patient reaching Complete Remission, 2 reaching Partial remission and 5 experiencing proteinuria reduction) at this time-period. However, extrapolating from Table 1 of source article 1, average proteinuria returned to baseline levels at 12 months. It should be noted 5 patients stopped treatment by 6 months.
Lopez-Martinez, 2022	Budesonide	5	NR	Enteric Budesonide	Nil	Proteinuria reduction of: - 26.7% at 3 months - 50.0% at 12 months - 15.3% at 24 months
Gandolfini, 2023	Budesonide	10	NR	TR-Budesonide (Ferring Pharmaceuticals), 2–9 mg daily for up to 6 months	Nil	No significant change in proteinuria and continued deterioration in graft function (-4.11 ml/min/1.73m^2^ per year average)
Cordeiro Cabral, 2018	Statin	19	NR	Any	Other subgroups	No effect in univariate analysis

Abbreviations: NR, not reported; RAAS, renin angiotensin aldosterone system; SC, subcutaneous; TR, targeted release

**Table 5:** Summary of management categories, number of interventions, availability of comparators, reported outcomes, considered evidence strength and main limitations

Intervention Category	Reported exposures	Comparator Availability	Reported Outcomes	Evidence Strength	Main Limitations
RAAS-Blockade	399, 11 studies [15, 16, 18, 19, 22, 27, 30, 38–41]	Limited	Proteinuria reduction and stabilisation of allograft function in some cohorts. Results were mixed.	Low-moderate	Poor dosing data; Conflicting results
Baseline Immunosuppression Adjustments	78, 5 studies [17, 18, 21, 22, 30]	Limited	No signal of benefit	Very low	Heterogeneous changes, often with overlapping therapies and incomplete dosing data Small subgroup numbers
Corticosteroid-based Strategies	Oral corticosteroid escalation reported in 100 exposures across 4 studies [28, 30, 31, 39]; Pulsed corticosteroids reported in 94 exposures across 9 studies [20–22, 26–28, 30, 31]. Categories overlapped.	Limited	Proteinuria reduction reported in some studies, often in conjunction with overlapping therapies. Outcomes were poor in RPGN cohorts despite therapy	Very low	Often pulsed corticosteroids reserved for more severe phenotypes; Maintenance steroid dose often not disclosed; Multiple overlapping therapies limits meaningful interpretation
Rituximab	32, 3 studies [32, 37, 39]	Limited	Proteinuria reduction and improved graft function / survival compared to controls	Low	Small, selected cohorts; 2 studies (3 and 21 patients) from same author group, overlap and centre details not explicitly reported; Other study included overlapping therapies.
Cyclophosphamide	8, 1 study [30]	Limited	No benefit in a univariate analysis	Very low	Single study with small numbers, multiple overlapping therapies and confounders
Complement Inhibition	5, 1 study [35]	No	Proteinuria reduction in some patients; repeat biopsy showed reduced complement deposition, but clinical responses were variable.	Very low	Single case series, used as a rescue treatment
BAFF / APRIL Inhibition	10, 1 study [36]	No	Initial proteinuria reduction but rebounded at 12 months.	Very low	Single case series, 5 patients stopped treatment early
Budesonide	15, 2 studies [33, 34]	No	Mixed results – no effect in one study, proteinuria reduction in one study (still present at 24 months)	Very low	NefIgArd targeted-release formulation not used; Dosing regimens unclear; Conflicting results
Tonsillectomy	52, 6 studies [23–27, 29]	Limited	Resolution of dipstick abnormalities, reduction in proteinuria, stabilisation of graft function, histological improvement on repeat biopsy	Low	Geographical restriction (Japan) without external validity; Higher rates of protocol biopsy with less severe phenotypes; Overlap with pulsed corticosteroids in 2 studies

Abbreviations: RAAS-blockade; Renin-angiotensin-aldosterone-system-blockade, BAFF; B-cell activating factor, APRIL; a proliferation-inducing ligand

Patients frequently received multiple therapies; therefore, intervention categories are not mutually exclusive. Evidence strength reflects a qualitative author assessment and should not be interpreted as a formal evaluation, consistent with standard scoping review methodology

Reported outcomes are outlined in Table 4. A summary of the available evidence for each intervention category, including overall evidence strength and key limitations, is provided in Table 5. Key outcomes such as proteinuria and graft function were not consistently reported. A variety of outcome measures were utilised. Follow-up was often short, thus limiting measurements and reporting of graft survival.

Three studies investigated histological changes following intervention [26, 29, 35]. Although sample sizes were small, the repeat histological assessment did provide additional mechanistic insights.

Follow-up duration and subgroup graft function / proteinuria are available in Supplementary Table 6 (where reported).

#### RAAS-Blockade

RAAS-Blockade was the most frequently reported management strategy described in 11 studies including 399 patients (21 received dual RAAS-blockade, 121 received RAAS-blockade alongside beta-blockers or calcium channel blockers). Several studies reported proteinuria reduction [15, 27, 38] or improved graft survival [16, 40] with RAAS-blockade. Ganesh et al. [39] observed proteinuria reduction with RAAS-blockade but in combination with corticosteroids. Conversely, four studies did not observe clear graft-related benefit [18, 22, 30, 41].

Park et al. [40] compared single agent RAAS-blockade, single agent beta-blocker / calcium channel blocker, and combination therapy. Of these three groups, single agent RAAS-blockade was associated with the most favourable death-censored graft survival, while combination regimens were associated with more severe clinical features. Cordeiro Cabral et al. [30] included a subgroup of 21 patients who received dual RAAS-blockade with no graft survival benefit demonstrated in multivariate analysis. Courtney et al. [19] reported higher rates of graft survival in post-transplant IgAN patients treated with RAAS-blockade; the actual graft survival measurements in each group were not provided, thereby limiting interpretation.

Reporting of drug type and dose was limited with only two studies specifying the agents used [15, 38], and only one study providing dosage [38] preventing determination of the number of patients in receipt of the maximally tolerated therapy, or disentanglement of overlapping treatment regimens.

#### Adjustment to baseline immunosuppression

Five studies (78 interventions, which may overlap in individual patients) described adjustment to baseline immunosuppression, including: switching azathioprine to a mycophenolic acid derivative (*n* = 25); addition of a mycophenolic acid derivative (*n* = 6); switching ciclosporin to tacrolimus (*n* = 5); addition of azathioprine (*n* = 2); unspecified (*n* = 40). Interventions often overlapped with other therapies. Overall, the evidence of efficacy was limited, with most studies not observing any meaningful benefit in outcomes. Interpretation is further constrained by small subgroup sizes and incomplete dosing data.

#### Corticosteroid-based strategies

Corticosteroid-based strategies were difficult to summarise because oral escalation, IV pulsed methylprednisolone, and subsequent oral maintenance frequently overlapped.

Nine studies (94 patients) described use of intravenous ‘pulses’ of corticosteroids. Two studies (23 patients) documented a subsequent regimen of oral corticosteroids [28, 31]. Many studies involved additional concurrent interventions such as RAAS-blockade [30, 39], other immunosuppression adjustment [21, 22, 30], rituximab [39], apheresis [39], and tonsillectomy [26, 27]. Indication for pulsed corticosteroids varied, although crescents on biopsy were the most common trigger. In cohorts with crescentic RPGN, outcomes were invariably poor with most grafts failing within the first year of follow-up [20, 21]. Two studies [28, 31] reported reductions in proteinuria following pulsed corticosteroids with an oral maintenance regimen, but whether this translated into improved graft survival was not demonstrated. Overall, no consistent therapeutic benefit emerged, constrained by small sample sizes, overlapping therapies, lack of comparators and clinical heterogeneity.

Oral corticosteroid escalation with RAAS-blockade was reported in 38 patients by Ganesh et al. [39]. A reduction in proteinuria was observed, but as therapies overlapped it is impossible to discern the individual benefit of either treatment.

Oral corticosteroid escalation was also reported in 39 patients by Cordeiro Cabral et al. [30]. In this study, multiple overlapping interventions were reported, including RAAS-blockade, cyclophosphamide, pulsed corticosteroids, and baseline immunosuppression adjustment. Individual patient treatments were not provided, and overlap is therefore hard to discern. Cox regression was used to address this; cumulative oral corticosteroid dose was associated with an increased risk of graft loss in a univariate analysis (*p* = 0.004), however this association became non-significant in a multivariate analysis (*p* = 0.4).

#### Rituximab

Three studies (32 patients) described favourable outcomes with anti-cluster of differentiation 20 (CD20) therapy using rituximab. Chancharoenthana et al. reported proteinuria reduction and improved allograft outcomes in two cohorts (*n* = 3 and *n* = 21) without corticosteroids [32, 37]. In the larger series, patients treated with rituximab had superior proteinuria reduction, improved allograft function and better long-term allograft survival compared to 43 contemporaneous controls receiving standard care (RAAS-blockade to target blood pressure < 130/80mmHg). Ganesh et al. described 8 patients treated with rituximab [39], all of whom also received pulsed corticosteroids, with half undergoing apheresis, making attribution of the observed reduction in proteinuria to any one therapy impossible.

#### Cyclophosphamide

Use of cyclophosphamide (an alkylating agent) was reported in only one study involving 8 patients [30]. Treatment subgroups overlapped substantially and univariate analysis demonstrated no therapeutic benefit.

#### Complement inhibition

One small case series (5 patients) reported the use of iptacopan (a small molecule complement factor B inhibitor) as rescue therapy after other interventions had failed [35]. Three patients had an improvement in proteinuria by 6 months but this was sustained in only one patient by 12 months and graft function deteriorated in 2 patients despite treatment. Repeat biopsies performed 6–18 months after therapy demonstrated reduction in both C3 and C5b-9 deposition indicating effective complement inhibition although clinical responses were variable.

#### BAFF/APRIL inhibition

A single study (10 patients) evaluated telitacicept (a dual inhibitor of the cytokines B cell activating factor – BAFF - and a proliferation-inducing ligand - APRIL) in post-transplant IgAN [36]. Patients received 160 mg telitacicept by weekly subcutaneous (SC) injection, escalating to 240 mg weekly on a case-by-case basis. Five patients completed the full 12-month treatment. Proteinuria reductions were observed at 3, 6 and 9 months but had rebounded to baseline at 12 months; one patient developed rejection at this time point.

#### Budesonide

Two studies (15 patients) described enteric budesonide use. Gandolfini et al. described 10 patients treated with budesonide [34]. The reported regimen was 9 mg for 6 months; however, supplement data suggests patients received varying doses (of 2–9 mg) and treatment durations. No patient demonstrated any proteinuria improvement. Graft function still deteriorated (-4.11 ml/min/1.73m^2^ per year). The variable dosing regimens limit treatment fidelity and make interpretation of the null findings difficult.

Conversely, Lopez-Martinez et al. reported on five patients treated with an unspecified formulation of budesonide with proteinuria reductions of 26.7% at 3 months and 50.0% at 1 year [33]. At 2 years, proteinuria remained 15.3% below baseline. Neither study used the targeted release budesonide formulation investigated in the NefIgArd trial [43].

#### Tonsillectomy

Six studies (52 patients), all from Japan, reported the use of tonsillectomy with eleven patients also receiving pulsed corticosteroids [26, 27]. Tonsillectomy was offered after the diagnosis of post-transplant IgAN and the individuals who declined tonsillectomy served as natural comparators in several cohorts. Beneficial graft-related outcomes were reported, including resolution of dipstick urinalysis abnormalities [23, 25], reduction in proteinuria [24, 26, 27], and histological improvements on repeat biopsy [26, 29]. In two studies with explicit comparator, outcomes favoured tonsillectomy over conservative management [24, 29].

### Safety

Adverse outcomes were rarely explicitly reported – only 12 studies specifically reported on measured adverse outcomes, but recording varied. Any escalation of therapy in immunosuppressed transplant recipients carries additional infective risk. Generally, where reported, only minor infections were noted. However, one study reported on higher infective complications (40% of total cohort, including 1 death) which was considered secondary to an escalation of immunosuppression [30]. Any modification to immunosuppressive therapies also carries an increased risk of graft rejection – this was only explicitly reported in two patients, one receiving budesonide [34] and one receiving telitacicept [36]. Adverse outcomes are available in Supplementary Table 6.

## Discussion

To our knowledge, this is the first scoping review specifically mapping diagnostic criteria, post-diagnosis management strategies, and reported outcomes in post-transplant IgAN. The scoping review intentionally focussed on management after diagnosis, rather than prevention of recurrence. Consequently, 27 studies met the inclusion criteria. Available literature is limited, consisting primarily of small retrospective single-centre cohorts and case series.

Marked heterogeneity in the definition of post-transplant IgAN was observed. Some cohorts defined post-transplant IgAN solely based on mesangial IgA deposition, whereas other studies required the presence of glomerular changes and/or accompanying clinical features to make a diagnosis. Debate persists as to the relevance of isolated IgA deposition in otherwise stable transplant recipients. Haas et al. surveyed pathologists and nephrologists and concluded that 71% of pathologists regarded IgA deposition alone as sufficient for a diagnosis of recurrent IgAN, compared to 25% of nephrologists. Nephrologists put more emphasis on additional histological features (i.e. cellular proliferation) and clinical features [44].

Detailed clinical and histological features were inconsistently reported across studies, limiting the assessment of disease severity and thus comparability between cohorts. Included populations likely represented a spectrum of disease ranging from incidental IgA deposition on protocol biopsy, to clinically significant disease associated with proteinuria, allograft dysfunction, and proliferative histological lesions. Some case series reported on RPGN. This heterogeneity complicates the interpretation of treatment effects, with differences in disease activity and treatment thresholds likely influencing observed outcomes.

RAAS-blockade was most frequently used and associated with benefit in several observational cohorts. Tonsillectomy may be beneficial in Japanese populations but has not been validated outside of Japan. The observed effects in rituximab were of interest; however, interpretation is limited by small sample size, concurrent therapies, and non-randomised study designs. A small randomised controlled trial (RCT) in native IgAN did not demonstrate efficacy despite B-cell depletion [45]. Further prospective studies are required to validate the efficacy of rituximab for post-transplant IgAN.

Recent case series assessed emerging therapies including iptacopan, telitacicept and budesonide. These novel agents are entering clinical practice in native IgAN; however, usage is limited in many healthcare settings due to the significant associated treatment costs. Further analysis will be required to establish the long-term efficacy of these treatments.

There was a paucity of evidence to guide adjustment of baseline immunosuppression or cyclophosphamide. Mixed outcomes were observed with pulsed corticosteroids, which may relate to overlapping interventions and significant heterogeneity. In studies that included RPGN, outcomes were invariably poor.

This review is timely as we are entering a new paradigm in native IgAN management, with multiple strategies targeting the mechanistic pathways implicated in this disease. These include suppressing galactose-deficient-IgA1 production – targeted-release Budesonide [43], BAFF/APRIL inhibitors [46–49]; complement inhibition – factor B inhibition [50], terminal C5 inhibition [51]; modifying downstream fibrosis – dual endothelin A / angiotensin receptor blockade [52, 53]; targeting dysregulated haemodynamics – sodium-glucose cotransporter 2 (SGLT2)-inhibitors [54, 55].

Despite these advances, post-transplant IgAN remains excluded from large clinical trials, creating an important evidence gap. Of note, no post-transplant studies including sparsentan or SGLT2-inhibitors met pre-specified inclusion criteria. An RCT is currently recruiting for sparsentan (NCT07219121, registered 9th October 2025). These treatments have proven efficacy in native IgAN, and thus exploring their benefit in transplant recipients is an urgent need.

### Ongoing future clinical trials

**Table 6:** Summary of currently ongoing clinical trials which include post-transplant IgAN in the kidney allograft

Trial Registration	Treatment	Status	Registration	Last Updated	Comments
ChiCTR2500114867, ChiCTR (via ICTRP)	Nefecon	Recruitment pending	18/12/2025	05/01/2026	Kidney transplant recipients with histologically confirmed IgAN in kidney allograft
NCT07219121, Clinicaltrials.gov	Sparsentan	Recruiting	09/10/2025	27/02/2026	Includes post-transplant IgAN and FSGS
NCT06983028, Clinicaltrials.gov	Atacicept	Recruiting	13/05/2025	20/01/2026	Multiple autoimmune glomerular diseases, but includes post-transplant IgAN
ChiCTR2500096335, ChiCTR (via ICTRP)	Telitacicept	Recruitment pending	22/01/2025	27/01/2025	Kidney transplant recipients with histologically confirmed IgAN in kidney allograft
ChiCTR2400089399, ChiCTR (via ICTRP)	Telitacicept	Recruitment pending	08/09/2024	17/09/2024	Kidney transplant recipients with histologically confirmed IgAN in kidney allograft
ChiCTR2400084654, ChiCTR (via ICTRP)	Telitacicept	Recruitment pending	22/05/2024	27/05/2024	Kidney transplant recipients with histologically confirmed IgAN in kidney allograft
NCT02571842, Clinicaltrials.gov	Rituximab	Unknown	06/10/2015	08/10/2015	Unknown status

Abbreviations: ChiCTR; Chinese Clinical Trial Register, ICTRP; International Clinical Trials Registry Platform, IgAN; IgA Nephropathy, FSGS; Focal Segmental Glomerulosclerosis

Two clinical trial registries (Clinicaltrials.gov, WHO International Clinical Trials Registry Platform) were examined for ongoing trials in post-transplant IgAN. Seven trials were identified, which are outlined in Table 6. Six specifically target transplant recipients with post-transplant IgAN, while one includes both native and post-transplant IgAN. Two studies also include other glomerular diseases. Investigational approaches include targeted-release budesonide (Nefecon), sparsentan, and B-cell therapies including atacicept, telitacicept and rituximab. Most trials remain in recruitment or pending recruitment, and outcome data are not yet available. Collectively, these studies suggest a growing interest in evaluating emerging therapies for post-transplant IgAN.

### Implications for clinical practice

There is a paucity of high-quality evidence to guide the management of post-transplant IgAN. There may be a rationale for supportive therapies including RAAS-blockade. Unfortunately, specific evidence supporting modification or addition of enhanced immunosuppression remains insufficient. There may be a role for high-dose pulsed steroids, rituximab, and novel therapies such as budesonide, telitacicept and iptacopan. Tonsillectomy could be considered in Japanese patients, but there is a lack of evidence to support its use outside of Japan.

### Strengths and limitations

To our knowledge, this is the first scoping review specifically mapping diagnostic criteria, post-diagnosis management strategies, and reported outcomes across 27 studies in post-transplant IgAN. JBI/PRISMA-ScR methodology was utilised with input from a medical librarian. Comprehensive extraction and charting were performed.

Substantial heterogeneity was observed, both with respect to diagnostic criteria and outcome definitions. This complicates the interpretation of treatment effects and limits the ability to pool outcomes across cohorts. In addition, adverse events were rarely explicitly reported. As a result of significant heterogeneity, scoping review methodology is ideal to map the current evidence.

Several limitations should be acknowledged. Exclusion of conference abstracts and English-language restriction may have resulted in the omission of emerging or preliminary data, particularly given the rapidly evolving therapeutic landscape in IgAN. Full-text screening and extraction was performed by a single reviewer following a proportionate calibration screen. While this pragmatic approach reflected the exploratory nature of the review, it may have introduced selection or extraction bias. Consistent with standard scoping review methodology, formal bias assessment was not undertaken.

Publication and indication bias are also important considerations when interpreting findings. Positive outcomes may be overrepresented within the literature, given the predominance of small observational studies and case series. Furthermore, treatment was inherently non-randomised, with some cohorts reserving more aggressive therapies for clinically severe phenotypes (for example, proteinuria thresholds or histological findings such as crescents or endocapillary proliferation). Thus, observed outcomes may reflect disease severity and treatment selection rather than a true treatment effect. Several studies included protocol biopsies, which may reflect less severe disease.

Another limitation to consider is the predominance of male recipients. Outcomes in larger cohorts were often not sex-segregated, and therefore it was not possible to discern if response varies between male and female recipients. Furthermore, the inability to reliably distinguish recurrent from *de novo* IgAN represents a further limitation. Consequently, it was not possible from the eligible studies to discern if response varied between these two potentially distinct entities post-transplantation.

## Conclusion

We are entering a new era in IgAN management, however evidence in post-transplant IgAN remains limited. Significant heterogeneity exists between diagnostic criteria, treatment strategies, and reported outcomes. Whilst supportive therapies including RAAS-blockade are commonly used, there is no evidence to support modification of baseline kidney transplant immunosuppression. Some additional immunosuppressive therapies (such as pulsed corticosteroid, rituximab) may be beneficial, but this evidence is limited to small observational studies. Novel therapies (non-targeted release of budesonide, telitacicept, iptacopan) are being utilised, but larger prospective multicentre studies are required.

Collectively, these findings underscore the absence of a standardised management pathway for post-transplant IgAN and highlight substantial gaps in the evidence base. There is now a clear need for the nephrology and transplant communities to deliver adequately powered, prospective, multicentre trials with harmonised diagnostic criteria and outcome measures to define optimal, evidence-based care for this high-risk population.

## Supplementary Information

Below is the link to the electronic supplementary material.

Supplementary Material 1

## Data Availability

The datasets used and/or analysed during the current study are available from the corresponding author on reasonable request.

## Abbreviations

Aza: Azathioprine
APRIL: A proliferation-inducing ligand
BAFF: B-cell activating factor
eGFR: Estimated glomerular filtration rate
ESKD: End-stage kidney disease
Gd-IgA1: Galactose-deficient IgA1
IFTA: Interstitial fibrosis and tubular atrophy
IgAN: Immunoglobulin A nephropathy
JBI: Joanna Briggs Institute
MEST(-C): Oxford classification system for assessing immunoglobulin A nephropathy histologically. This includes M: Mesangial hypercellularity, E: Endocapillary proliferation, S: Segmental sclerosis/adhesion, T: Tubular atrophy/ interstitial fibrosis, C: Crescents;
MMF: Mycophenolate mofetil
MPA: Mycophenolic acid derivative
NR: Not reported
OSF: Open Science Framework
PCR: Protein:creatinine ratio
RAAS: Renin-angiotensin aldosterone system
RCT: Randomised controlled trial
RPGN: Rapidly progressive glomerulonephritis
SC: Subcutaneous
TR: Targeted-release
