# Supplementary material for: Management strategies for post-transplant IgA nephropathy in kidney transplant recipients: a scoping review
Source: BMC Nephrol. 2026 Aug 20;27:517. doi: 10.1186/s12882-026-05260-x (PMC13540840; doi:10.1186/s12882-026-05260-x)
Supplement: Supplementary file 1 [file 12882_2026_5260_MOESM1_ESM.docx]

**Scoping Review – Supplement**

**Supplementary Table 1: MEDLINE ALL Search**

| 1.  2.  3.  4.  5.  6.  7.  8.  9.  10.  11.  12.  13.  14.  15.  16.  17.  18. | Glomerulonephritis, IGA/  (IgA adj3 (glomerul* or nephr* or disease or mesangial)).mp.  ("immunoglobulin a" adj3 (glomerul* or nephr* or disease or mesangial)).mp.  (berger* adj1 (disease or syndrome or nephr*)).mp.  IgAN.mp.  1 or 2 or 3 or 4 or 5  Kidney Transplantation/  (kidney adj1 transplant*).mp.  (renal adj1 transplant*).mp.  (kidney adj1 (graft* or allograft*)).mp.  (renal adj1 (graft* or allograft*)).mp.  7 or 8 or 9 or 10 or 11  Recurrence/  recur*.mp.  relaps*.mp.  13 or 14 or 15  6 and 12 and 16  limit 17 to (English language and yr="2000 -Current") |
| --- | --- |

**Supplementary Table 2: Embase Search**

| 1.  2.  3.  4.  5.  6.  7.  8.  9.  10.  11.  12.  13.  14.  15.  16.  17.  18.  19.  20. | Immunoglobulin A nephropathy/  (IgA adj3 (glomerul* or nephr* or disease or mesangial)).mp.  ("immunoglobulin a" adj3 (glomerul* or nephr* or disease or mesangial)).mp.  (berger* adj1 (disease or syndrome or nephr*)).mp.  IgAN.mp.  1 or 2 or 3 or 4 or 5  Kidney graft/  (kidney adj1 transplant*).mp.  (renal adj1 transplant*).mp.  (kidney adj1 (graft* or allograft*)).mp.  (renal adj1 (graft* or allograft*)).mp.  7 or 8 or 9 or 10 or 11  Recurrent disease/  recur*.mp.  relaps*.mp. or relapse/  13 or 14 or 15  6 and 12 and 16  limit 17 to (english language and yr="2000 -Current")  conference abstract.pt. or exp conference paper/  18 not 19 |
| --- | --- |

**Supplementary Table 3: Web of Science Core Collection Search**

**"IgA Nephropathy" OR "IgAN" OR ("IgA" NEAR/3 (glomerul* OR nephr* OR disease OR mesangial)) OR ("immunoglobulin a" NEAR/3 (glomerul* OR nephr* OR disease OR mesangial)) OR (berger* NEAR/1 (disease OR syndrome OR nephr*))** (Topic) and **(kidney NEAR/1 transplant*) OR (renal NEAR/1 transplant*) OR (kidney NEAR/1 (graft* OR allograft*)) OR (renal NEAR/1 (graft* OR allograft*))** (Topic) and **recur* OR relaps*** (Topic) not **Meeting Abstract OR Meeting** (Document Type) and **English** (Languages)

**Supplementary Table 4: Scopus Search**

( TITLE-ABS-KEY ( "IgA Nephropathy" OR "IgAN" OR ( "IgA" W/3 ( glomerul* OR nephr* OR disease OR mesangial ) ) OR ( "immunoglobulin a" W/3 ( glomerul* OR nephr* OR disease OR mesangial ) ) OR ( berger* W/1 ( disease OR syndrome OR nephr* ) ) ) AND TITLE-ABS-KEY ( ( kidney W/1 transplant* ) OR ( renal W/1 transplant* ) OR ( kidney W/1 ( graft* OR allograft* ) ) OR ( renal W/1 ( graft* OR allograft* ) ) ) AND TITLE-ABS-KEY ( recur* OR relaps* ) ) AND PUBYEAR > 1999 AND ( EXCLUDE ( DOCTYPE , "cp" ) ) AND ( LIMIT-TO ( LANGUAGE , "English" ) )

**Supplementary Table 5: Additional Baseline Characteristics at time of transplant (for patients with post-transplant IgAN)**

| Study | Age at Transplant | Male (%) | Female (%) | Living Donor (%) | Deceased Donor (%) | Immunosuppression |
| --- | --- | --- | --- | --- | --- | --- |
| Oka 2000 | NR | 76.2% | 23.8% | NR | NR | Not reported at time of transplant. At time of diagnosis (for intervention group (n=10): CsA and steroids (60%), Tac, Aza and steroids (20%), Aza and steroids (20%) |
| Jeong 2003 | NR | 77.8% | 22.2% | 100% | 0 | CsA based double or triple immunosuppression reported as baseline |
| Namba 2004 | NR | 75% | 25% | 87.5% | 12.5% | Not reported at time of transplant. At time of diagnosis, 83% on a CsA based regimen, 17% on a Tac based regimen |
| Jeong 2004 | NR | 76.1% † | 23.9% † | 100% † | 0% † | Patients only included if they received CsA based double immunosuppression (not otherwise specified) |
| Chandrakantan 2005 | NR | NR | NR | 60% | 40% | Not reported specifically for post-transplant IgAN cohort. In all patients transplanted (regardless of recurrence), a CNI based triple therapy regimen was utilised. 60% were on MMF, 40% on Aza |
| Courtney 2006 | NR | 85.3% | 14.7% | NR | NR | Maintenance immunosuppression regimens evolved over study period and included Aza, prednisolone, CsA (after 1989), MMF (after 1998) and Tac (after 2002).  Breakdown not provided at time of diagnosis. |
| Mousson 2007 | NR | NR | NR | 0% | 100% | Various immunosuppressive regimens utilised over 15-year study period, including ATG, corticosteroids, CNIs, Aza, or MMF |
| Tang 2008 | NR | 80% | 20% | 0% | 100% | Until September 1997: 8 patients, administered CsA, Aza, and prednisolone  After October 1997: 2 patients, administered CsA, MMF, and prednisolone |
| Kiattisunthorn 2008 | 35.2 ± 11.2 | 80% | 20% | 50% | 50% | Not reported specifically at time of transplant, but at time of diagnosis CsA and prednisolone (56.7%), CsA, prednisolone and Aza (30%), CsA, prednisolone and MMF (6.7%), prednisolone and MMF (3.3%) |
| Ushigome 2009 | NR | 50% | 50% | 100% | 0% | CsA based triple therapy with either Aza (pre-2002) or MMF (post-2002) |
| Kennoki 2009 | NR | 60.7% * | 29.3% * | 100% | 0% | Tac based triple therapy with MMF and prednisolone |
| Koshino 2013 | 34.1 + 9.7 | 42.9% | 57.1% | 100% | 0% | CsA based triple therapy (with either mizoribine or mycophenolate) at time of transplant |
| Hotta 2013 | NR | 60% | 40% | NR | NR | Tac based triple therapy with MMF and prednisolone |
| Wang 2014 | 44.0 + 12.9 | 40% | 60% | 80% | 20% | NR |
| Messina 2016 | 45.8† | 93.1% | 6.9% | 17.2% | 82.8% | 25 patients received baseline CNI based therapy with steroids with or without MMF  4 patients received baseline mTORi with steroids and either low dose calcineurin inhibitor or MMF. 55% of patients had discontinued steroids at time of diagnosis. |
| Chancharoenthana 2017 | NR | 100% | 0% | 100% | 0% | At time of diagnosis, CsA and MMF (n=1), mTORi and MMF (n=1), CsA monotherapy (n=1) |
| Nihei 2017 | 35.4† | 66.7% | 33.3% | 91.7% | 8.3% | CNI based triple therapy with Tac / CsA, mycophenolate / Aza, and methylprednisolone (no breakdown) |
| Cordeiro Cabral 2018 | 34 + 9.6 | 68.1% | 31.9% | 85.1% | 14.9% | CNI based triple therapy with steroids and either: Aza (70.2%), MPA (19.1%), mTOR inhibitor (8.5%), or fingolimod (2.1%) |
| Matsukuma 2018 | NR | 71.4% | 28.6% | 85.7% | 14.3% | CNI based triple therapy with steroids and either MMF (71.4%) and Aza (28.6%) |
| Park 2019 | NR | 67%† | 33%† | 80.4%† | 19.6%† | Not provided at time of transplant, at time of diagnosis 48.5% on Tac, 93.3% on steroids, 71.1% on MPA |
| Chancharoenthana 2021 | NR | 57.8% | 42.2% | 92.2% | 7.8% | CNI based triple therapy (97% Tac) with MMF and steroids |
| Uffing  2021 | 41 (32-54) | 73.2% | 26.8% | 49% | 51% | Reported immunosuppression consisted of Tac, MMF and steroids (73%), CsA, MMF and steroids (15%), Tac and MMF (5%), other (7%) |
| Lopez-Martinez 2022 | NR | 100% | 0% | NR | NR | NR |
| Gandolfini 2023 | NR | 90% | 10% | 50% | 50% | All on Tac and steroids with either MMF (70%), mTORi (10%), Aza (10%) or nothing additional (10%) |
| Ganesh 2024 | 45 + 11.25 | 79.7% | 20.3% | NR | NR | Tac based triple therapy with MMF and steroids |
| Sanna 2025 | 43.8† | 60% | 40% | Not fully reported: n=1 | Not fully reported: n=3 | All patients initially received standard immunosuppression regimens which included Tac, MMF, and prednisolone |
| Xu 2025 | NR | 60% | 40% | NR | NR | Typically included Tac with MMF (or sodium mycophenolate), alongside steroids |

† weighted averages from provided data

* provided only for intervention and control subgroups

Abbreviations: CsA, ciclosporin; Tac, tacrolimus; Aza, azathioprine; MMF, mycophenolate mofetil; MPA, mycophenolic acid; ATG, anti-thymocyte globulin; mTORi, mammalian target of rapamycin inhibitor; NR, not reported

**Supplementary Table 6: Characteristics of intervention / control subgroups and adverse outcomes (where provided)**

| Study | Intervention / Subgroup | Baseline Proteinuria | Baseline Creatinine or *eGFR* | Follow-up after therapy | Proteinuria (last follow-up) | Creatinine or *eGFR* (last follow-up) | Adverse Outcomes Reported during follow-up |
| --- | --- | --- | --- | --- | --- | --- | --- |
| Oka 2000 | Trandalopril (n=10) | 2.7 g/day | NR individually | 12.8 mo | 0.89 g/day | NR | NR |
| Jeong 2003 | Enalapril (n=7) with Segmental Sclerosis | 2.41 g/day | 1.44 mg/dL | 39.3 mo | 2.00 g/day | 3.11 mg/dL | Presence of segmental sclerosis predicted poorer reduction in proteinuria and higher incidence of graft dysfunction (creatinine increment >100% in 3 of 7 cases) |
|  | Enalapril (n=7) without Segmental Sclerosis | 1.8 g/day | 1.20 mg/dL |  | 1.19 g/day | 1.49 mg/dL |  |
| Namba 2004 | Any RAAS-Blockade (n=18) | NR individually | NR individually | 110.9 mo | NR | NR | NR |
| Jeong 2004 | Addition of MMF (n=2) | NR individually | NR individually | NR | NR | NR | NR |
|  | Addition of Aza (n=2) | NR individually | NR individually | NR | NR | NR | NR |
| Chandrakantan 2005 | Any RAAS-Blockade (n=13), no comparator | NR individually | NR individually | NR individually | NR | NR individually | NR |
|  | Switching Aza to MMF (n=9) | NR individually | NR individually | NR individually | NR | 1.4–3.0 mg/dL (n=1)  >3.0 mg/dL (n=1)  End-stage kidney disease (n=7) | NR |
|  | No change to immunosuppression (n=11) | NR individually | NR individually | NR individually | NR | < 1.4 mg/dL (n=3)  1.4-3.0 mg/dL (n=1)  >3.0 mg/dL (n=2)  End-stage kidney disease (n=5) | NR |
| Courtney 2006 | Any RAAS-Blockade (n=9) | NR | NR | NR | NR | NR | NR |
|  | No RAAS-Blockade (n=4) | NR | NR | NR | NR | NR | NR |
| Mousson 2007 (16) | Pulsed Steroids (n=2) | NR individually | NR | 2 and 27 mo | NR | NR | NR |
| Tang 2008 | Pulsed Steroids (n=10) | 2.06g g/day (n=8) | 3.37 mg/dL | 6-60 mo | NR | Failed (n=9)  Static 2.5 mg/dL (n=1) | NR |
|  | Switching CsA to Tac (n=5) | 1.65 g/day (n=3) † | 3.1 mg/dL † |  | NR | Failed (n=4)  Static 2.5 mg/dL (n=1) | NR |
|  | Switching Aza to MMF (n=5) | 2.51 g/day (n=4) † | 2.91 mg/dL † |  | NR | Failed (n=5) | NR |
| Kiattisunthorn 2008 | RAAS-blockade (n=13) | NR individually | NR individually | 36 mo | NR individually | NR individually | NR |
|  | Pulsed Steroids (n=2) | NR individually | NR individually |  | NR individually | NR individually | NR |
|  | Addition of MMF (n=2) | NR individually | NR individually |  | NR individually | NR individually | NR |
|  | Switching Aza to MMF (n=2) | NR individually | NR individually |  | NR individually | NR individually | NR |
| Ushigome 2009 | Tonsillectomy (n=4) | NR | 1.85 mg/dL | 13.5 mo | NR | 1.75 mg/dL | NR |
| Kennoki 2009 | Tonsillectomy (n=16) | 0.88 g/day | 1.2 mg/dL | 12 mo | 0.28 g/day | 1.2 mg/dL | No surgical complications |
|  | No Tonsillectomy (n=12) | 0.68 g/day | 1.3 mg/dL |  | 0.76 g/day | 1.5 mg/dL |  |
| Koshino 2013 | Tonsillectomy (n=7) | Dipstick 0-3+ | 1.65 mg/dL | 48.4 mo | Dipstick 0-3+ | End-stage kidney failure (n=2),  Figures otherwise non-extractable | NR |
| Hotta 2013 | Tonsillectomy (n=7) | NR individually | NR individually | 23.8 mo | NR individually | NR individually | No surgical complications |
|  | Tonsillectomy and Pulsed Steroids (n=8) | NR individually | NR individually |  | NR individually | NR individually |  |
| Wang 2014 | Any RAAS-Blockade (n=5), no comparator | 0.78 g/day | NR | 6 mo | 0.40 g/day | NR | NR |
|  | Tonsillectomy (n=2) | 0.4 g/day (no individual breakdown) | NR | 45.4 mo | 0.06 g/day (no individual breakdown) | NR | NR |
|  | Tonsillectomy and Pulsed Steroids (n=3) |  | NR |  |  | NR | NR |
| Messina 2016 | Pulsed Steroids with maintenance (n=16) | 1.65 g/day | 1.9 mg/dL | 45.6 mo | 0.9 g/day | 1.8 mg/dL | NR |
|  | No escalation of steroid therapy (n=13) | 1.7 g/day | 1.9 mg/dL | 64.8 mo | 1.9 g/day | 2.7 mg/dL | Specified no adverse effects |
| Chancharoenthana 2017 | Rituximab (n=3) | 4.79 g/day † | 1.25 mg/dL † | 26 mo | NR | NR | Flu-like illness (n=1) |
| Nihei 2017 | Tonsillectomy (n=5) | 0.33 g/g Cr | *47.7 ml/min/1.73m^2^* | 60 mo | 0.20 g/g Cr | *34.8 ml/min/1.73m^2^* | NR |
|  | No Tonsillectomy (n=7) | 0.07 g/g Cr | *39.1 ml/min/1.73m^2^* |  | 0.97 g/g Cr | *47.7 ml/min/1.73m^2^* | NR |
| Cordeiro Cabral 2018 | Statin (n=19) | NR Individually | NR Individually | 42.5 mo | NR Individually | NR Individually | Authors highlight infections occurred in 40% during or after immunosuppression therapy: Pulmonary infections (20%)  Urinary tract infections (10%)  Herpes zoster (5%)  Cytomegalovirus (5%)  Skin infection (5%)  Fungal infection (5%)  Sepsis unknown source (2.5%)  1 death due to septic shock |
|  | Immunosuppression Change (n=40) | NR Individually | NR Individually |  | NR Individually | NR Individually |  |
|  | Oral Steroids (n=39) | NR Individually | NR Individually |  | NR Individually | NR Individually |  |
|  | Pulsed Steroids (n=30) | NR Individually | NR Individually |  | NR Individually | NR Individually |  |
|  | Cyclophosphamide (n=8) | NR Individually | NR Individually |  | NR Individually | NR Individually |  |
|  | Switching Aza to Mycophenolate (n=9) | NR Individually | NR Individually |  | NR Individually | NR Individually |  |
|  | Any RAAS-Blockade (n=42) | NR Individually | NR Individually |  | NR Individually | NR Individually |  |
|  | Dual RAAS-Blockade (n=21) | NR Individually | NR Individually |  | NR Individually | NR Individually |  |
| Matsukuma 2018 | Pulsed Steroids with maintenance (n=7) | 0.82 g/g Cr | *48.7 ml/min/1.73m^2^* | 24 mo | 0.26 g/g Cr | *47.4 ml/min/1.73m^2^* | Herpes zoster (n=1) |
| Park 2019 | Any RAAS-Blockade as monotherapy (n=38) | 1.5 mg/dL | Dipstick 0- (23.7%) 1+ (21.1%)  2+ (55.3%) | 60 mo | NR | NR | NR |
|  | CCB or β-Blocker without RAAS-Blockade (n=33) | 1.9 mg/dL | Dipstick 0- (60.6%) 1+ (21.2%)  2+ (18.2%) |  | NR | NR | NR |
|  | Combination RAAS-Blockade with CCB and/or β-Blocker (n=121) | 1.7 mg/dL | Dipstick 0- (35.0%) 1+ (14.2%)  2+ (50.8%) |  | NR | NR | NR |
|  | No additional therapies (n=272) | 1.7 mg/dL | Dipstick 0- (69.7%) 1+ (10.0%)  2+ (20.3%) |  | NR | NR | NR |
| Chancharoenthana 2021 | Rituximab (n=21) | 4.51 g/day | 2.0 mg/dL | 45.6 mo | NR | NR | Specific analysis of adverse events on an intention-to-treat basis. No infusion reactions or serious adverse events reported. |
|  | Control (n=43) i.e. maximal RAAS-Blockade to achieve BP <130/80 | 4.04 g/day | 2.1 mg/dL |  | NR | NR |  |
| Uffing  2021 | Any RAAS-Blockade (n=78) | NR | NR |  | NR | NR | Associated post-transplant adverse events associated with IgAN recurrence collected, but not attributed to therapies |
|  | No RAAS-Blockade (n=30) | NR | NR |  | NR | NR |  |
| Lopez-Martinez 2022 | Budesonide (n=5) | 1.5 g/day | NR | 24 mo | 0.75 g/day (12 mo)  1.27 g/day (24 mo) | NR | Specifically monitored HbA1c, BMI and LDL which remained stable. No adverse events reported. |
| Gandolfini 2023 | Budesonide (n=10) | NR | NR | NR | NR | NR | Graft failure (n=3), Rejection (n=1), Diabetes mellitus (n=1), bacterial infection (n=1), viral infection (n=3), fungal infection (n=1), muscle pain (n=2).  No psychiatric symptoms |
| Ganesh 2024 | Rituximab with Pulsed Steroids (n=4) | 2.32 g/day (no individual breakdown) | 1.48 mg/dL (no individual breakdown) | 12 mo | 1.05 g/day (no individual breakdown) | 1.68 mg/dL (no individual breakdown) | NR |
|  | Rituximab with Pulsed Steroids and Plasma Exchange (n=4) |  |  |  |  |  |  |
|  | Increase in oral steroids, any RAAS-Blockade (n=38) | 1.11 g/day † | 1.83 mg/dL † |  | 0.55 g/day † | 1.71 mg/dL † |  |
| Sanna 2025 | Iptacopan (n=5) | 2.36 g/day † | 1.88 mg/dL † | 5-19 mo | 1.64 g/day † (6 mo, n=5)  0.9 g/day † (12 mo, n=3) | 2.59 mg/dL † (6 mo, n=5)  2.59 mg/dL † (12 mo, n=3) | Transient discontinuation due to infection (n=2) but both able to restart therapy. No safety concerns identified. |
| Xu 2025 | Telitacicept (n=10) | 2.92 g/day | 141.9 µmol/L | 12 mo | 1.96 g/day (6 mo)  3.11 g/day (12 mo) | 151.5 µmol/L (6 mo)  154.3 µmol/L (12 mo) | Discontinuation of treatment due to lack of response (n=3) and financial constraints (n=2)  White cell counts monitored – stable neutrophils, elevated lymphocytes (45.25% from baseline). ALT and Bilirubin remained stable.  Diarrhoea (n=1)  Upper respiratory tract infection (n=1)  Urinary tract infection (n=1)  Nausea and dizziness after injection, relieved spontaneously (n=2)  Antibody mediated rejection (n=1) |

† Weighted averages from provided data

Abbreviations: RAAS, renin-angiotensin-aldosterone system; CsA, ciclosporin; Tac, tacrolimus; Aza, azathioprine; MMF, mycophenolate mofetil; MPA, mycophenolic acid; ATG, anti-thymocyte globulin; mTORi, mammalian target of rapamycin inhibitor; CCB, calcium-channel blocker; eGFR, estimated glomerular filtration rate; g/g Cr, grams per gram creatinine; Dipstick 0-, none or trace; HbA1c, glycated haemoglobin; BMI, body mass index; LDL, low-density lipoprotein; ALT, alanine aminotransferase; mo, months; NR, not reported; NR individually, subgroup not reported individually (but combined figures represented in manuscript table 2)
